# Solving the mystery of the yellow zone of the asthma action plan

**DOI:** 10.1038/s41533-017-0067-1

**Published:** 2018-01-11

**Authors:** Samir Gupta, Alan Kaplan

**Affiliations:** 1 Department of Medicine, Division of Respirology, University of Toronto, Toronto, Canada; 2 The Keenan Research Centre in the Li Ka Shing Knowledge Institute of St. Michael’s Hospital, Toronto, ON Canada; 3 University of Toronto, Toronto, Canada; 4 Family Physician Airways Group of Canada, Edmonton, Canada

Asthma presents a paradigm for the benefits of self-management, more than any other chronic disease. This is due to both the rapid and unpredictable nature of asthma worsenings and the remarkable ability for inhaled anti-inflammatory medications to mitigate these worsenings.

This self-management is operationalized through a written asthma action plan (AAP)—a simple piece of paper with a “green zone” describing good asthma control and reinforcing baseline medications, a “yellow zone” describing acute loss of control and corresponding instructions for therapeutic intensification, and a “red zone” indicating severe symptoms prompting immediate medical assistance.^1^ The functional principle of this tool is simple: if patients quickly intensify therapy when their asthma starts to worsen, they can avert a full-blown flare and the need for urgent healthcare and systemic corticosteroids.

Throughout the 1990s, this intuitive concept was put to the test in a series of randomized-controlled trials (RCTs). In 2000, and again in 2003, Gibson and colleagues systematically reviewed these data in a Cochrane review of 18 RCTs, concluding that use of a written AAP in conjunction with education and regular clinical review significantly reduces hospitalizations, emergency room visits, unscheduled visits to the doctor, number of days off work or school, and nocturnal asthma symptoms, and significantly improves quality of life.^2^ Accordingly, as early as 1996,^3^ asthma guidelines across the world recommended that each asthma patient should receive an AAP.

Yet over 20 years later, use of AAPs remains a niche practice, and a glaring example of ineffective respiratory guideline implementation. Only 29% of patients received an AAP in a 2001 Australian study,^4^ and 23% in a 2006 UK report.^5^ More recent data are even more disappointing, with only 4% of surveyed Canadian primary care physicians reporting consistently providing a written AAP,^6^ and only 2% of Canadian^7^ and American^8^ patients having actually received one. Although this problem has mostly been reported in primary care, where the majority of asthma patients are seen, AAP delivery remains below 50% even in tertiary care centers.^9^

So what went wrong? Primary care barriers to AAP delivery have been well-described. Some barriers have to do with the AAPs themselves. Our analysis of 69 AAPs collected from prior RCTs and existing asthma programs across the world demonstrated large variability in both their content and format, and poor usability.^10^ Most plans were developed ad-hoc, and by content experts exclusively. Other barriers exist at the level of providers, the practice environment, and the overall health care system. Qualitative studies indicate that a majority of physicians consider AAPs to be important, but fail to provide them due to lack of time.^11^ In addition, physicians are limited by lack of experience and confidence in generating appropriate AAP recommendations, lack of confidence in their patients’ ability to utilize them,^12^ and lack of their availability at the point of care.^11,13,14^ In one study, 30% of physicians attending an asthma skills workshop were unable to prepare an adequate AAP, with the main knowledge gap surrounding how to change therapy in the yellow zone of the AAP.^14^

In turn, this knowledge gap may be driven by poor guidance. Primary care physicians complain that guidelines are too lengthy, ambiguous, and complex, and are presented in too rigid a fashion for practical application in individual patients.^15^ Our recent analysis identified corresponding concerns with the “implementability” of several guidelines.^15^ Although the most recent Canadian Asthma Guideline (2012) attempts to address this knowledge gap by providing evidence-based recommendations for changes to therapy in the yellow zone of the AAP, this complex process remains challenging to operationalize.

In order to try to address these knowledge and usability barriers, our group sought to develop a practical, evidence-based, point-of-care guide for populating adult AAP yellow zone instructions. To achieve this, we started with a review of AAP guidance found in major asthma guidelines published in the last five years (including the Global Initiative for Asthma (GINA), British Thoracic Society/Scottish Intercollegiate Guideline Network (BTS/SIGN), and Canadian Thoracic Society (CTS) guidelines). We supplemented this with a systematic literature search for relevant reports published more recently. Based on the synthesis of these data, we established evidence-based rules for changes to therapy in the AAP yellow zone. Next, we tested the applicability of these rules across common baseline controller medication dose and frequency regimens in Canada, USA, and Europe. As expected, we discovered several operational challenges in applying these recommendations. In some cases, guidelines provided no clear approach. In others, the universal recommendation to increase ICS dosing by 4–5 fold in the yellow zone could not be applied because dosing would exceed jurisdictional regulatory dose limits. These issues affected 15 of 43 (35%) common European dose regimens; however we were able to identify and recommend alternate evidence-based approaches in 8 of these 15 (53%) circumstances.

Dose increases in the AAP yellow zone can also be achieved in a variety of ways, including changes to the number and/or frequency of inhalations, through addition of a new inhaler, or through temporary replacement of the baseline medication with a more potent inhaler. Again, guidelines did not offer practical advice on how dose increases should be achieved. To address this, we established basic principles for formulating yellow zone prescriptions that sought to maximize patient satisfaction and adherence while minimizing patient errors, according to the best evidences available, and expert opinion where evidence was lacking.

This work was published in the European Respiratory Journal on May 1st, 2017.^16^ The freely accessible publication includes easy-to-follow, printable, paper-based algorithms that we hope clinicians will post in their clinical settings, to inform completion of the AAP yellow zone (one for each of Europe, Canada, and the US, in Appendix 1 of the publication). We believe that this tool will help to address what has been described as clinicians’ need for “practical evidence-based advice about how to select and construct the most effective and appropriate action plan for all of their patients.”^17^ We also hope that this work can be adopted as an implementation tool across international guidelines, enabling harmonizing of care.

However, we acknowledge that this tool only addresses knowledge, which is one of several barriers to AAP delivery. Successful broad-scale AAP implementation will likely require patient and clinician education, improved communication, and ideally, shared decision-making. Other enablers would include prompting by patients, ensuring that AAPs are available at the point of care, and allied health support for AAP review.^6^ At the same time, patient-directed interventions will be required to maximize actual patient use of AAPs. Our group has attempted to address many of these needs through the Electronic Asthma Management System (eAMS)—a tool which enables clinicians to automatically generate a personalized AAP based on patient inputs in a pre-visit electronic questionnaire and clinician inputs in an electronic medical record-integrated decision support system. Results of a clinical trial of this system will soon be available.

More broadly, it is also important to note that self-management AAPs must be reviewed regularly and accompanied by patient education in order to have their desired effects. In fact, although also limited by knowledge and time barriers, the importance of asthma education as part of the larger structured review required for successful asthma management should not be underestimated. This includes ensuring that objective testing has confirmed the asthma diagnosis, particularly given that asthma is often “over-diagnosed” and erroneously labeled patients may face harms from unnecessary pharmacotherapy.^18^ There is also a need to regularly evaluate adherence to both trigger avoidance and pharmacotherapy, and to utilize targeted adherence interventions.^19^ Similarly, clinicians should provide practical advice to optimize inhaler technique.^20^ Finally, all current smokers should be counseled to quit at each clinical interaction.

Although great strides have been made in asthma therapy over the last few decades, asthma still kills. Given their unequivocal benefit, our collective failure to consistently provide our patients with AAPs is a likely contributor. Experts note that the persistent “lack of clearly-defined protocols for action plans is a significant disincentive for their use.”^17^ Accordingly, we hope that our protocolized approach to determining instructions for the AAP yellow zone will prove an important first step in empowering primary care physicians to increase their use of AAPs.
